# Synchrony within, synchrony without: establishing the link between interpersonal behavioural and brain-to-brain synchrony during role-play

**DOI:** 10.1098/rsos.240331

**Published:** 2024-09-24

**Authors:** Mengyu Lim, Alessandro Carollo, Andrea Bizzego, Annabel S. H. Chen, Gianluca Esposito

**Affiliations:** ^1^ Psychology Program, School of Social Sciences, Nanyang Technological University, Singapore; ^2^ Department of Psychology and Cognitive Science, University of Trento, Trento, Italy

**Keywords:** synchrony, fNIRS, behaviour, role-play, empathy, anxiety

## Abstract

Interpersonal synchrony is a crucial construct in understanding social interactions, which has been used in clinical studies to measure the quality of the therapeutic alliance. However, there is a lack of studies investigating the correlation between synchrony expressed on different levels: behavioural and neurophysiological. Furthermore, there are no studies that examine how the implementation of psychodramatic role-playing techniques, when individuals adopt the persona of a different character, may influence intrinsic biobehavioural synchrony between two parties. The present study, therefore, aims to uncover the relationship between behavioural and brain-to-brain synchrony across different role-playing techniques and elucidate the impact of these synchronies on participants’ levels of anxiety and empathy. By using functional near-infrared imaging and behavioural coding in a dyadic role-playing paradigm (*n* = 41 dyads), the study found correlations between behavioural and brain-to-brain synchrony during naturalistic conversations, but not during role-play, implying a qualitative change in interpersonal synchrony when implementing role-playing techniques. Additionally, the study noted significant contributions of both behavioural and brain-to-brain synchrony as well as peripheral factors such as dyadic sex make-up and role immersion in predicting dyadic anxiety and empathy changes. Findings call for future studies to consider role-playing scenarios as a qualitatively different form of social interaction.

## 1. Introduction

As human beings, we are social creatures who are continuously shaped by the nature and quality of social interactions occurring around us. These social interactions are bi-directional; multiple parties influence each other and create a unique dynamic that can only be observed within the individuals sharing the interaction. One pivotal feature of social interaction is the emergence of interpersonal synchrony, defined as the phenomenon of interpersonal coordination, attunement or co-regulation over time [1]. Since its first conceptualization, the construct of interpersonal synchrony has been applied to understanding the behaviours and social dynamics of various relational groups (e.g. family [2]; friends [3]; lovers [4] and even strangers [5,6]) and teamwork contexts (e.g. in terms of music-making [7] or coordinated sports [8]). In these studies, it has been found that interpersonal synchrony is a key contributor to rapport building [6,9], empathy [10] and emotion regulation [2], with implications for team performance [7,8]. Interpersonal synchrony can be measured in a variety of ways, such as by capturing similarities in movement and posturing, or by computing similarities in physiological signals. This results in different types of synchrony reported across studies, which will be subsequently explored.

In fact, it is only recently that studies have moved beyond an exclusively behavioural approach to synchrony to incorporate hormonal, physiological and even neural information (e.g. behavioural with brain-to-brain synchrony in [11,12]), a trend that is of particular relevance when attempting to enhance the interpretability of synchrony findings across studies [13]. In the clinical context, understanding relationships between different levels of synchrony is even more crucial. The notion of behavioural synchrony assisting in the establishment of brain-to-brain synchrony is the bedrock of applied synchrony in clinical contexts and the theorized reason behind how synchrony can allow the client and clinician access to the other’s internal states, enabling understanding and emotional sharing [14].

Therefore, the investigation of synchrony has been extended to clinical contexts [14] as a means of characterizing the therapeutic alliance [15] beyond the joint pursuit of shared clinical goals [16]. In this case, the establishment of interpersonal synchrony in real-time is related to building clinician–client rapport, enhancing attunement to the internal states of the client, and is unique between pairs of clinicians and clients [17]. Interpersonal synchrony in clinical studies has been measured predominantly with behavioural variables [16]. In fact, these studies have found that behavioural synchrony between client and clinician is critical in understanding the quality of the therapeutic alliance and in predicting client dropout [18]. It is thought that high levels of synchrony between the therapist and client are indicative of therapist empathy [10,19], with implications on the client’s outcomes [20]. For example, Ramseyer & Tschacher [17] found that increased client–clinician behavioural synchrony was related to a greater reduction in the symptom and a higher client self-efficacy. However, clinical studies employing synchrony also operationalize synchrony in a myriad of ways. While these studies show that synchrony between individuals can be observed across multiple layers of the self, ranging from neurological [21] and physiological (e.g. hormone levels [20]; heart rate and respiration [4,22]; skin conductance [19]) to behavioural (e.g. vocal arousal [10]; synchrony in movement [17]), these differences may cause difficulties in interpreting and integrating results across different modalities of measurement. Depending on the modality used, synchrony measurements vary in temporal resolution and implicated processes. The study by Zilcha-Mano *et al*. [20], for example, measured oxytocin changes of both clinician and client over 16 intervention sessions, implicating whole-of-body regulation through hormonal changes in the long term, while other available literature tended to investigate synchrony within discrete sessions using measures of higher temporal resolution (e.g. heart rate [4,22] and brain activation [21]), implicating comparatively localized processes in the shorter term.

This is particularly true when considering clinical role-playing activities. Role-playing is commonly implemented as part of psychotherapy owing to its ability to grant its participants the opportunity to practise coping strategies and alternative ways of responding that are discussed during clinical interventions [23], as well as the opportunity for situational or personal reappraisal, particularly as clients embody different personas or traits different from their own [24,25]. Role-playing, particularly in the clinical context, is commonly associated with improvements in empathy [26] and a reduction in social anxiety [27,28]. For example, Dogan’s work [26] showed that role-playing activities facilitate empathy by decreasing defensiveness and increasing the closeness and depth of the therapeutic relationship. On the other hand, participants in the study [28] demonstrated decreases in self-reported anxiety and an improvement in social skills as a result of role-playing activities.

Influences of role-play in psychotherapy can be traced back to psychodrama, which espouses the use of role-playing techniques to dramatically re-enact scenarios from clients’ lives in a group setting [29]. From psychodrama, a variety of role-playing techniques, such as role reversal (where two people role-play as each other [29,30]) were introduced and adapted for implementation across other modes of psychotherapy [31]. Cognitively, role-play is a demanding activity that necessitates the maintenance of at least two personas (i.e. the self and the role being played) but only the expression of a single persona at any one time. The adoption of different personas when using different role-playing techniques has been shown to relate to systematic differences in brain activity as compared to natural conversations [32] and holds interesting implications in interpersonal synchrony. Specifically, we have found in a previous study a deactivation in the anterior left prefrontal cortex [32], suggestive of an inhibition of self-referential networks during role-play. Non-clinical role-playing neuroimaging investigations also showed similar patterns of deactivation [33]. However, findings thus far are limited to the level of the individual, and there are presently limited studies that investigate how brain-to-brain synchrony may be changed during role-play. Despite a lack of empirical evidence, the theory of psychodramatic role-play does take into consideration the effect of bi-directional influences when multiple individuals role-play simultaneously, alluding to the construct of interpersonal synchrony as we know it today. For example, in the sociocognitive model of role reversal, a common role-playing technique used in psychodrama, the third and final phase of role feedback refers to the constant adaptation of each individual to their role-playing partner as they perceive not only their partner’s role-playing behaviour but also how their partner responds and adjusts to their own role-playing [34]. In other words, the co-regulation present during role reversal involves looking at oneself through another’s eyes [29].

### 1.1. The present study

Given the proliferation of role-playing activities making use of various role-play techniques in modern psychotherapy [31], it is important to understand how role-play influences the intrinsic interpersonal co-regulation that is already taking place. Further, the embodiment of another role during role-play implies that the individual is maintaining multiple identities or personas during a role-play session, but is only expressing the behaviour and affect of a single character. There may be yet uncovered relationships between multiple facets of interpersonal co-regulation, particularly when considering behavioural and neurological systems. Furthermore, the adoption of other personas during role-play techniques may affect these relationships between behavioural and brain-to-brain synchrony. Taking together the literature presented above, the present study set out to (i) investigate the association between brain-to-brain and behavioural synchrony between dyads when different role-playing techniques are implemented, and (ii) connect these relationships to broader clinical outcomes; specifically, the impact of interpersonal synchrony across role-playing techniques on dyadic empathy and anxiety will be investigated. Two main research questions were posed:

— RQ1: can brain-to-brain synchrony during different role-playing conditions be predicted by behavioural synchrony?
— RQ2a: can significant changes in the dyad’s empathy before and after the experimental session be predicted by neuro-behavioural synchrony during the role-play conditions? and
— RQ2b: can significant changes in the dyad’s anxiety before and after the experimental session be predicted by neuro-behavioural synchrony during the role-play conditions?

While there are no preceding studies that inform the hypotheses to the research questions posed above, particularly with regard to the different role-play conditions, the extant literature suggests that we will observe significant correlations between behavioural synchrony with brain-to-brain synchrony and that both behavioural and brain-to-brain synchrony will contribute significantly to the dyads’ change in empathy and anxiety across the session.

## 2. Material and methods

### 2.1. Participants

Participants were 41 pairs of friends (*n* = 82; 24 female–female, 6 female–male, 11 male–male) recruited from the undergraduate research participation pool of the School of Social Sciences, Nanyang Technological University and social media networks using a combination of convenience and snowball sampling. All participants were healthy adults residing in Singapore, a nation in Southeast Asia, within the age range of 18 to 35 years (mean = 21.95, s.d. = 3.11). Exclusion criteria included the presence of any known or diagnosed medical or psychological disorder. Depending on their source of recruitment from the University or external avenues respectively, participants were reimbursed with S$10 or three academic credits each as a token of appreciation for their participation. The study protocol and materials have been approved by the Institutional Review Board of Nanyang Technological University (IRB-2021-03-013).

### 2.2. Material and equipment

#### 2.2.1. Overall anxiety severity and impairment scale

The overall anxiety severity and impairment scale (OASIS [35]), a non-clinical measure of anxiety severity and its associated impairment in various areas of function, consists of five items, each rated on a five-point Likert scale. Psychometric analyses revealed that OASIS has good internal reliability, as well as construct, convergent and discriminant validities [35]. In this study, both pre- and post-session OASIS had adequate Cronbach’s alphas of 0.87.

#### 2.2.2. Interpersonal reactivity index

The interpersonal reactivity index (IRI [36]), a measure of empathy, consists of 28 items, each rated on a five-point Likert scale. IRI can also be broken down into four subscales, namely fantasy, empathic concern, perspective taking and personal distress. The consideration of empathy evoked in fictitious contexts through the fantasy subscale was particularly relevant to the investigation of role-play where the scenarios are not executed in real life. Psychometric analyses revealed that IRI has good internal and test–retest reliabilities, as well as concurrent, construct, convergent, discriminant, incremental and predictive validities [36,37].

As the focus of the study was on empathic change towards the participants’ role-playing partner as a result of the experimental conditions, the items were adapted according to Péloquin & Lafontaine [37] to measure empathy specifically within the participant dyad instead of others, in general. In this study, pre- and post-session IRI had Cronbach’s alpha of 0.69 and 0.85, respectively.

#### 2.2.3. Role-playing character sheet

Participants identified characters to role-play during the experimental conditions and completed a hardcopy form containing open-ended prompts. These prompts contained questions related to their chosen character’s personality and behaviour, inspired by Collier & Collero’s model [38] on learning role-related behaviour which emphasizes the understanding and association of role behaviour with the self. The questions posed to the participants are as follows:

— Q1: how long have you known this person?
— Q2: how/where did you come to know about this person?
— Q3: what is this person like in terms of personality? and
— Q4: how does this person usually behave when interacting with another friend?

In addition, for the third and fourth questions, participants were encouraged to answer these questions in first-person perspective (i.e. ‘I am’ instead of ‘he/she is’), in alignment with the protocol designed by Brown *et al*. [33].

#### 2.2.4. Self-rated role-playing experience

Upon the completion of role-play and role reversal conditions, a total of three questions were presented to the participants. These questions measured the participants’ perceived (i) relatability of their chosen character, (ii) accuracy of their portrayal of the selected character, and (iii) immersion during the condition. Participants provided their ratings on a five-point Likert scale.

#### 2.2.5. McGill friendship questionnaire

The McGill friendship questionnaire (MFQ [39]), a measure of friendship quality and specifically the respondents’ affection towards their peers, consists of 16 items, each rated on a nine-point Likert scale. Psychometric analyses revealed that MFQ has good internal reliability [39]. In this study, the MFQ had a Cronbach’s alpha of 0.95.

#### 2.2.6. Functional near-infrared spectroscopy hyperscanning

The prefrontal cortical activity of all participants was measured using the hyperscanning functional near-infrared spectroscopy (fNIRS) technique (NIRSport, NIRx Medical Technologies LLC) on NIRStar software (v. 15.2, Windows 64-bit). The prefrontal cortex configuration is a standardized set-up incorporating eight sources and seven detector optodes, forming a total of 20 channels akin to the international 10–20 system used in electroencephalography (EEG) [40]). To detect relative changes in blood oxygenation level, 760 nm and 850 nm wavelengths were used [41] at a sampling rate of 7.81 Hz. The above fNIRS set-up has been used earlier in related studies investigating prefrontal cortical activity during dyadic interactions (e.g. [2,42]).

To aid the interpretation of fNIRS signals beyond discrete channels, and in line with the shift towards regional network imaging [43], the fNIRS channels were further aggregated during the data pre-processing stage into four anatomical clusters of the prefrontal cortex (i.e. frontal left and right, medial left and right), similar to past work by Lim *et al*. [32] and Azhari *et al*. [2,44]. More details regarding the clustering of fNIRS signals are provided in §3.4.1.

#### 2.2.7. Video camera

Videos of the three experimental conditions were obtained with a Sony HDR-PJ240E model digital video camera recorder. The video camera was mounted on a tripod that was positioned approximately 2 m away from the participants. Videos captured participants’ whole bodies.

### 2.3. Experimental protocol

Participants were first screened for their eligibility and invited to complete an online pre-laboratory questionnaire containing the OASIS, IRI, as well as basic demographic details including participants’ age, biological sex and the duration of their friendship with each other. Additionally, a binary yes/no question regarding participants’ past experience with role-playing activities was posed. Therefore, for each dyad, both participants could report having prior experiences with role-play. Alternatively, only one participant within the dyad may have such experiences, or none of them may report prior experience. Finally, when both participating members of the recruited dyad had completed the questionnaire, the laboratory session was arranged.

The within-subjects experimental protocol consists of three experimental conditions (naturalistic conversation, role-play and role reversal) which are counterbalanced for each dyad. Each condition lasted 5 min. As with all within-subjects designs, the scenario presented to participants for each condition was identical, and the only manipulation was the change in role-play technique. In naturalistic conversation, participants acted as themselves; in role-play, participants selected another mutually known friend, classmate or colleague each and in role reversal, participants acted as each other. Participants were invited to take a seat on chairs arranged at 45° to each other, with the video camera positioned approximately 2 m away facing them. The fNIRS set-up was calibrated to maximize signal quality, or until 15 min had elapsed, before commencing the study. Hyperscanning fNIRS and video data were recorded during each condition. Prior to the role-play and role reversal conditions, as participants were no longer acting as themselves, the role-playing character sheets were completed. The scenarios for each experimental condition were communicated to the participants using a printed placard and by reading aloud the instructions ad verbatim.

Specifically, the scenarios required participants to imagine themselves (or their selected personas) at a shopping mall buying a gift for each other. This task was adapted from a related study on mentalizing by Kanske *et al*. [45] and was chosen as it did not intentionally elicit negative emotions that may alter the nature and eventual interpretation of interpersonal synchrony. When selecting scenario prompts, the activity of gift giving is found to be relatable across different cultures [46,47], compared with other activities such as a trip to the cinema or overseas travel, and therefore, had the highest validity when considering the generalizability of results.

Self-rated responses concerning participants’ experiences of the role-play and role reversal conditions were obtained after the conclusion of the respective conditions. At the end of all three conditions, participants were invited to complete the OASIS and IRI again as a post-session measure, as well as the MFQ. Finally, participants were reimbursed for their time, thanked and debriefed.

### 2.4. Data analysis plan

For both fNIRS and video data, only the middle 3 min (corresponding to the second, third and fourth minute of each condition) were preserved for analysis. This was to account for linguistic differences in the structure of a social interaction, as the beginnings and endings of a conversation tend to be different from its middle [48]. To create a dyadic value for questionnaire data, raw scores from each member of the dyad were totalled.

#### 2.4.1. Functional near-infrared spectroscopy pre-processing and synchrony analysis

The fNIRS pre-processing and synchrony analysis follow the procedure used in Lim *et al*. [49]. Raw fNIRS data were pre-processed with the *pyphysio* package [50] based on Python. Firstly, a trained machine learning model [51], based on convolutional neural networks, was used to evaluate the signal quality of each fNIRS channel. Next, motion artefacts were identified and corrected using spline interpolation [52] and wavelet filtering [53] in a two-step process [54]. Then, using the modified Beer–Lambert law, raw signals based on fNIRS wavelengths were converted to concentrations of oxygenated and deoxygenated haemoglobin (HbO and Hb, respectively). Signals were, subsequently, put through a third order, Butterworth infinite impulse response bandpass filter at 0.01–0.5 Hz [55].

**Figure 1.**
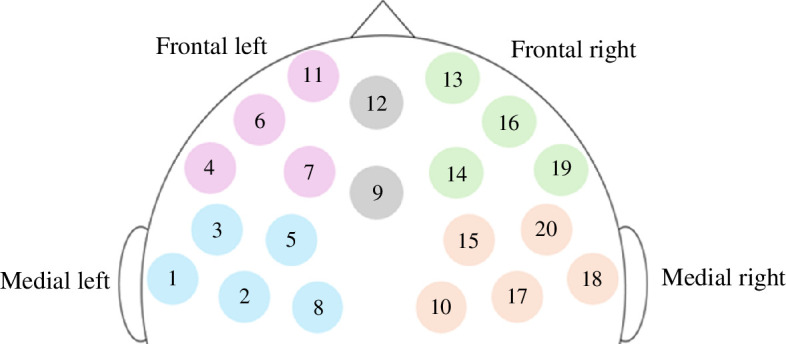
Graphical representation of the clustering scheme.

After pre-processing, HbO data were normalized and aggregated into four anatomical clusters in the prefrontal cortex. The channels and corresponding aggregated clusters are represented in table 1 and figure 1. Cluster data were only calculated if a majority of channels (i.e. three or more) representing the said cluster contained good signals after pre-processing. Otherwise, cluster data were not calculated and the fNIRS signals for the said cluster were discarded. Missing cluster values, together with missing questionnaire data owing to technical difficulties (corresponding to 1.48% of the dataset), were addressed by imputation.

**Table 1.** Anatomical clustering scheme for fNIRS channels. (In this scheme, channels 9 and 12 are not classified under any of the above clusters.)

cluster	channels
frontal left	4
	6
	7
	11
frontal right	13
	14
	16
	19
medial left	1
	2
	3
	5
	8
medial right	10
	15
	17
	18
	20

Time-series fNIRS data from each participant, after clustering, were then matched with their role-play partner. Brain-to-brain synchrony was computed based on these data using the wavelet transform coherence (WTC; [56]), only among homologous brain regions. The WTC measures the extent of similarity or shared oscillating patterns between two or more signals across various frequency bands and time intervals. The WTC has been previously used in related synchrony analyses involving physiological signals, including EEG and functional magnetic resonance imaging data [57–59]. In contrast with other methods of synchrony calculations such as cross-correlation, the WTC is especially suitable as it does not rely on a fixed frequency band and, therefore, more appropriately models naturalistic interactions where interpersonal patterns and rhythms may be unstable or vary across time [60,61].

Additionally, to determine if the synchrony coefficient obtained was higher than the chance level, a surrogate analysis was also adopted where time-series data of single participants were randomly matched with another member of a different participating dyad. Comparative analyses between true and surrogate dyads can then be conducted.

#### 2.4.2. Behavioural micro-coding

Behavioural micro-coding was conducted on Solomon Coder (v. beta 19.08.02, Windows 32-bit) at the same sampling frequency as fNIRS data (i.e. 7.81 Hz). Coding was conducted by a team of six trained undergraduate student assistants, who achieved an acceptable inter-rater agreement of 70.3% on 10% of the dataset. The training was conducted over three separate 1–2 h sessions and included the operation of the coding software, definition and operationalization of behavioural variables, as well as to reconcile differences in coding. Disagreements were solved with collaborative discussions and mutual agreement on the final coding.

Behaviours coded included instances of synchronous behaviour, such as joint gaze (participants directly gazing at each other), joint gesture (participants simultaneously employing communicative gestures such as handwaves, hand signals or body movement) and joint vocalizations of emotions (participants simultaneously laughing or vocalizing non-word sounds). These behaviours were selected owing to their relevance in social interactions based on previous frameworks on interpersonal communication [62,63], and have also been used in other studies investigating interpersonal synchrony [64–66]. However, it should be noted that other measures of non-verbal synchrony, such as interpersonal distance and the mirroring of body posture, could not be fully implemented in the present study owing to the fixed seating positions in the experimental set-up. The behaviours were operationalized as a proportion of time when they were displayed by the dyad across the 3 min interaction.

#### 2.4.3. Statistical analysis

All statistical analyses were performed on RStudio (v. 1.3.1093, Windows 64-bit) in R (v. 4.0.3).

Following the procedures in Reindl *et al*. [67], a preliminary comparative analysis between true and surrogate dyads was conducted for each cluster to determine the signals where synchrony was achieved above the chance level. A one-tailed Mann–Whitney *U*-test (true > surrogate) was used. Only clusters where synchrony for true dyads was significantly greater than surrogate dyads were advanced for subsequent analyses.

To address the first research question, for each experimental condition, a forward stepwise linear regression was used to identify potential predictors of brain-to-brain synchrony in each selected cluster. The candidate predictors include:

— behavioural synchrony variables such as joint gaze, joint gesture and joint vocalization during each experimental condition;
— self-rated role-play experience including role relatability, role-playing accuracy and immersion during role-play and role reversal conditions; and
— dyadic characteristics such as sex make-up, the presence of past role-playing experience and the duration and quality of friendship (measured by MFQ).

To address the second research question, an initial pre-post comparison was done to determine the dyads’ change in anxiety and empathy scores. Repeated measures Wilcoxon signed-rank tests were conducted. Next, for significantly changed anxiety and empathy scores and sub-scales, a forward stepwise linear regression was used to identify potential predictors of these changes. The candidate predictors include:

— brain-to-brain synchrony of selected clusters during each experimental condition;
— behavioural synchrony variables such as joint gaze, joint gesture and joint vocalization during each experimental condition;
— self-rated role-play experience including role relatability, role-playing accuracy and immersion; and
— dyadic characteristics such as sex make-up, the presence of past role-playing experience and the duration and quality of friendship (measured by MFQ).

For all instances of forward stepwise linear regression, candidate predictors were included at each step based on their *p*-values and the stopping rule was based on the model’s Akaike information criterion (AIC). For all statistical comparisons, Bonferroni correction was applied.

## 3. Results

### 3.1. Preliminary analysis

Table 2 describes the comparative analysis between true and surrogate dyads for each anatomical cluster, as well as overall fNIRS signals.

After Bonferroni correction, analysis proceeded only for the frontal right cluster (*U* = 5513, *p* = 0.003).

**Table 2.** Comparative analysis between true and surrogate dyads. (One-tailed comparison; Bonferroni correction applied over four clusters (*p *< 0.0125). All significant results are denoted with asterisks: **p < 0.01.)

**cluster**	**true**			**surrogate**			**Mann–Whitney *U***	*p*‐value
	*n*	mean	s.d.	*n*	mean	s.d.		
frontal left	124	0.473	0.043	122	0.465	0.040	6722	0.066
frontal right	120	0.478	0.042	116	0.462	0.041	5513	0.003**
medial left	70	0.461	0.039	72	0.455	0.041	2846	0.496
medial right	77	0.458	0.039	89	0.458	0.038	3346	0.398
all	392	0.469	0.042	400	0.461	0.040	69 164	0.003**

### 3.2. Predictors of brain-to-brain synchrony during role-play techniques

Tables 3 and 4 present generated final models of predictors of frontal right cluster synchrony during naturalistic conversation and role reversal. Surprisingly, no predictors were included in the final model for the role-play condition (*p* > 0.05). Variables that do not contribute significantly to the models’ AIC were not included in tables 3 and 4.

**Table 3.** Predictors of frontal right cluster synchrony during naturalistic conversation. (Dyad sex was dummy coded with female–female: 0. Prior role-playing experience was dummy coded with mixed (i.e. one member has prior experience while the other does not): 0. All significant results are denoted with an asterisk: *p < 0.05.)

predictor	beta	s.e.	*t*-value	*p*‐value
dyad sex				
male–female	0.02328	0.03061	0.760	0.4548
male	0.04407	0.01725	2.555	0.0177*
joint gaze	−0.08988	0.03982	−2.257	0.0338*
prior experience				
no	0.04064	0.03247	1.252	0.2233
yes	−0.01782	0.01447	−1.232	0.2303
joint gesture	0.28702	0.19404	1.479	0.1527
*F*_6,23_ = 3.057, *p* = 0.02386 Adj. *R* ^2^ = 0.2985				

**Table 4.** Predictors of frontal right cluster synchrony during role reversal. (Dyad sex was dummy coded with female–female: 0. All significant results are denoted with an asterisk: *p < 0.05.)

predictor	beta	s.e.	*t*-value	*p*‐value
role relatability	−0.012498	0.004835	−2.585	0.0155*
immersion	0.008668	0.004273	2.028	0.0525
dyad sex				
male–female	0.001176	0.022079	0.053	0.9579
male–male	−0.030106	0.013830	−2.177	0.0384*
*F*_4,27_ = 3.251, *p* = 0.0267 Adj. *R* ^2^ = 0.2251				

Significant predictors of frontal right cluster synchrony during naturalistic conversation include dyad sex make-up (male–male dyads positively predict brain-to-brain synchrony in comparison with male–female or female–female dyads; *β* = 0.04, s.e. = 0.02, *p* = 0.02) and the proportion of time spent in joint gaze (longer joint gaze negatively predicts brain-to-brain synchrony; *β* = −0.09, s.e. = 0.04, *p* = 0.03). The overall model explained 29.9% of the overall variance (*F*_6,23_ = 3.06, *p* = 0.02).

Significant predictors of frontal right cluster synchrony during role reversal include dyad sex make-up (male–male dyads negatively predict brain-to-brain synchrony in comparison with male–female or female–female dyads; *β* = −0.03, s.e. = 0.01, *p* = 0.04) and role relatability (greater relatability to the character being role-played negatively predicts brain-to-brain synchrony; *β* = −0.01, s.e., 0.005, *p* = 0.02). The overall model explained 22.5% of the overall variance (*F*_4,27_ = 3.25, *p* = 0.03).

### 3.3. Neurobehavioural predictors of dyadic anxiety and empathy changes

Table 5 summarizes comparative pre-post analyses of OASIS, IRI and IRI subscale scores. There are significant decreases in dyad anxiety (*W* = 534, *p* = 0.02) and significant increases in dyad empathy (*W* = 55, *p* = 3.03 × 10^−6^), specifically in the empathic concern (*W* = 29, *p* = 3.10 × 10^−7^) and perspective taking sub-scales (*W* = 70.5, *p* = 1.36 × 10^−5^). Therefore, subsequent analyses proceeded only to uncover predictors influencing the pre-post changes in OASIS, IRI and its empathic concern and perspective taking sub-scales.

Tables 6–9 detail the generated final models of predictors of change in dyadic anxiety and empathy.

**Table 5.** Repeated measures Wilcoxon signed-rank tests for OASIS and IRI. (Two-tailed comparison; Bonferroni correction applied over IRI subscales. All significant results are denoted with asterisks: *p < 0.05*;* *** *p* < 0.005.)

measure	pre		post		Wilcoxon	***p*** **‐value**
	mean	s.d.	mean	s.d.		
OASIS	8.63	4.78	7.05	4.69	534	0.02*
IRI	127.9	14.66	144.6	18.49	55	3.03 × 10^−6^***
- fantasy	32.12	7.59	34.54	8.63	257.5	0.03
- empathic concern	32.07	6.19	41.68	6.21	29	3.10 × 10^−7^***
- perspective taking	35.93	4.59	40.39	5.26	70.5	1.36 × 10^−5^***
- personal distress	27.73	6.04	27.95	8.02	291	0.70

**Table 6.** Predictors of decrease in dyad anxiety. (All significant results are denoted with asterisks: *p < 0.05; **p < 0.01.)

predictor	beta	s.e.	*t*-value	*p*‐value
Frontal Right synchrony during Role Reversal	58.59238	18.58247	3.153	0.003740**
role accuracy during Role-Play	1.05678	0.40041	2.639	0.013231*
joint gaze during Role Reversal	−16.49597	5.48508	−3.007	0.005398**
joint gaze during Role-Play	12.61514	5.08135	2.483	0.019074*
friendship quality	0.08787	0.04956	1.773	0.086708
*F*_5,29_ = 5.063, *p* = 0.00186 Adj. *R* ^2^ = 0.374				

**Table 7.** Predictors of increase in dyad empathy. (Dyad sex was dummy coded with female–female: 0. All significant results are denoted with asterisks: *p < 0.05; **p < 0.01.)

predictor	beta	s.e.	*t*-value	*p*‐value
dyad sex				
male–female	−16.55448	10.629	−2.051	0.0496*
male–male	−23.49882	8.516	−3.188	0.0035**
immersion during Role Reversal	6.06340	5.678	3.144	0.0039**
joint vocalization during Naturalistic Conversation	245.35788	102.68287	2.389	0.023848*
role accuracy during Role Reversal	−3.20905	1.69967	−1.888	0.069422
friendship duration	−0.07840	0.04576	−1.713	0.097692
*F*_6,28_ = 5.812, *p* = 0.0004959 Adj. *R* ^2^ = 0.4592				

**Table 8.** Predictors of increase in dyad empathic concern. (Dyad sex was dummy coded with female–female: 0. All significant results are denoted with asterisks: *p < 0.05; **p < 0.01; ***p < 0.005.)

predictor	beta	s.e.	*t*-value	*p*‐value
dyad sex				
male–female	−12.8305	3.7419	−3.429	0.00211**
male–male	−12.4616	2.2813	−5.463	1.13 × 10^−5^***
immersion during Role Reversal	3.8575	0.8125	4.748	7.16 × 10^−5^***
role accuracy during Role Reversal	−3.5277	1.0242	−3.444	0.00203**
joint gaze during Naturalistic Conversation	−16.9392	6.5753	−2.576	0.01628*
joint gaze during Role-Play	19.0608	5.9827	3.186	0.00385**
joint vocalization during Naturalistic Conversation	97.7988	47.8853	2.042	0.005180
character relatability during Role Reversal	1.8493	1.2027	1.538	0.13669
Frontal Right synchrony during Role-Play	−41.2947	28.3769	−1.455	0.15805
*F*_9,25_ = 5.91, *p* = 0.0001995 Adj. *R* ^2^ = 0.5652				

**Table 9.** Predictors of increase in dyad perspective taking. (All significant results are denoted with asterisks: *p < 0.05; **p < 0.01; ***p < 0.005.)

predictor	beta	s.e.	*t*-value	*p*‐value
friendship duration	−0.064	0.01396	−4.584	9.32 × 10^−5^***
joint vocalization during Naturalistic Conversation	97.75675	33.52011	2.916	0.00705**
Frontal Right synchrony during Naturalistic Conversation	−74.18823	20.82915	−3.562	0.00139**
Frontal Right synchrony during Role-Play	63.96229	24.38465	2.623	0.01415*
Frontal Right synchrony during Role Reversal	35.97512	21.29396	1.689	0.10265
joint vocalization during Role-Play	42.77464	24.65056	1.735	0.0941
friendship quality	0.07937	0.05863	1.354	0.18702
*F*_7,28_ = 4.707, *p* = 0.001482 Adj. *R* ^2^ = 0.4328				

Significant predictors of change in dyad anxiety after the laboratory session include the dyad’s frontal right brain-to-brain synchrony during role reversal condition (greater brain-to-brain synchrony positively predicts a decrease in dyad anxiety; *β* = 58.59, s.e. = 18.58, *p* = 0.004), as well as behavioural synchrony in terms of joint gaze during both role-play and role reversal conditions. Specifically, longer joint gaze during role-play condition positively predicts a decrease in dyad anxiety (*β* = 12.62, s.e. = 5.08, *p* = 0.02), while the same behaviour during the role reversal condition has the opposite direction of effect (*β* = −16.5, s.e. = 5.49, *p* = 0.005). Additionally, the greater dyad’s perceived accuracy of role portrayal during the role-play condition positively predicted the decrease in dyad anxiety (*β* = 1.06, s.e. = 0.4, *p* = 0.01). The overall model explained 37.4% of the overall variance (*F*_5,29_ = 5.063, *p* = 0.002).

Significant predictors of change in dyad empathy after the laboratory session include dyad sex make-up (male–male and male–female dyads negatively predict an increase in dyad empathy in comparison with female–female dyads; *β* = −23.5, s.e. = 8.52, *p* = 0.004 and *β* = −16.55, s.e. = 10.63, *p* = 0.05, respectively), dyad’s immersion during role reversal (greater immersion positively predicts an increase in dyad empathy; *β* = 6.06, s.e. = 5.68, *p* = 0.004) and the proportion of time spent in joint vocalization during naturalistic conversation condition (longer joint vocalizations positively predicts an increase in dyad empathy; *β* = 245.36, s.e. = 102.68, *p* = 0.02). The overall model explained 45.9% of the overall variance (*F*_6,28_ = 5.81, *p* = 0.0005).

Significant predictors of change in dyad empathic concern after the laboratory session include dyad sex make-up (male–male and male–female dyads negatively predict an increase in dyad empathy in comparison with female–female dyads; *β* = −12.46, s.e. = 2.28, *p* = 1.13 × 10^−5^ and *β* = −12.83, s.e. = 3.74, *p* = 0.002, respectively), dyad’s immersion and a perceived accuracy of role portrayal during role reversal (greater immersion positively predicts an increase in dyad empathy; *β* = 3.86, s.e. = 0.81, *p* = 7.16 × 10^−5^, while a greater perceived accuracy has the opposite direction of effect; *β* = −3.53, s.e. = 1.02, *p* = 0.002) and the proportion of time spent in joint gaze. Specifically, longer joint gaze during the role-play condition positively predicts an increase in dyad empathy (*β* = 19.06, s.e. = 5.98, *p* = 0.004), while the same behaviour during the naturalistic conversation condition has the opposite direction of effect (*β* = −16.39, s.e. = 6.57, *p* = 0.02). The overall model explained 56.5% of the overall variance (*F*_9,25_ = 5.91, *p* = 0.0002).

Significant predictors of change in dyad perspective taking after the laboratory session include the duration of the dyad’s friendship with each other (longer friendship duration negatively predicts an increase in dyad perspective taking; *β* = −0.06, s.e. = 0.01, *p* = 9.32 × 10^−5^), proportion of time spent in joint vocalization during naturalistic conversation condition (longer joint vocalizations positively predicts an increase in dyad empathy; *β* = 97.76, s.e. = 33.52, *p* = 0.007), and frontal right cluster synchrony during both naturalistic conversation and role-play conditions. Specifically, greater brain-to-brain synchrony during the naturalistic conversation condition negatively predicts an increase in the dyad perspective taking (*β* = −74.19, s.e. = 20.83, *p* = 0.001), while the same during the role-play condition has the opposite direction of effect (*β* = 63.96, s.e. = 24.38, *p* = 0.01). The overall model explained 43.3% of the overall variance (*F*_7,28_ = 4.71, *p* = 0.001).

## 4. Discussion

The present study posed two research questions. First, it sought to investigate the relationship between behavioural and brain-to-brain synchrony across different role-playing conditions, including naturalistic conversation, role-play and role reversal. Second, the study sought to investigate how behavioural and brain-to-brain synchrony contribute to changes in the dyad’s empathy and anxiety level after the role-play session. Preliminary checks found that only the frontal right cluster, covering the right lateralization of the anterior prefrontal cortex, part of the dorsolateral prefrontal cortex and the pars orbitalis of the inferior frontal cortex, demonstrated significantly higher levels of brain-to-brain synchrony compared with randomly paired signals. Furthermore, significant pre-post session changes in participants’ anxiety and empathy levels, specifically with regard to empathic concern and perspective taking, were also observed.

The frontal right cluster was the only cluster to show significantly greater brain-to-brain synchrony among true dyads compared with other clusters. The observation implies that there are unique patterns of co-regulation among true dyads going through the experimental conditions together, as compared to surrogate dyads who go through the same conditions but do not interact together. A key function implicated in this region has to do with prospective and working memory [68], as well as the theory of mind processes [69–71], which may be recruited during dyadic interaction as participants converse, particularly as participating dyads have to keep track of the conversation and access shared knowledge (implicating memory processes), as well as attribute intention to their role-play partners (implicating theory of mind). These aspects of social conversations, together with the dynamics of the interaction, may contribute to unique dyadic co-regulation.

To address the first research question, findings revealed that in naturalistic conversation (i.e. where individuals did not assume alternative personas), joint gaze was a significant negative predictor of brain-to-brain synchrony in the frontal right cluster of the prefrontal cortex. In this condition, findings were aligned with the theory posited by Koole & Tschacher [14], albeit in an unexpected direction. During naturalistic conversation, a larger proportion of time spent in joint gaze was negatively related to brain-to-brain synchrony in the frontal right cluster. In fact, this finding also contrasts with a previous study that found similarities in the activity of brain structures within this cluster when imitating hand movements [72]. However, it should be noted that interpersonal synchrony is not purely about imitation, but also takes into account the context of the interaction. Based on the available literature, explanations for the direction of this correlation may be put forth. Cultural studies investigating joint gaze have consistently shown that unlike their Western counterparts where eye contact is construed positively and as indicative of paying attention [73], participants from Asian cultures tend to find excessive eye contact rude [74], perceiving the individual making eye contact to be more unpleasant, angry and unapproachable [75]. The present study findings corroborate this phenomenon from a different direction, where greater joint eye contact is related to decreased interpersonal attunement at the brain level. In this study similarly involving all Asian participants, elevated eye contact may be employed as a compensatory mechanism for poor brain-to-brain synchrony when participants sought to understand their partner’s intentions and actions, particularly as joint gaze contributes to mentalization [76]. This is supported by previous findings that the frontal right cluster is associated with intention attribution and the theory of mind [69–71].

Interestingly, in the role-play and role reversal conditions, none of the behavioural measures of synchrony emerged as significant predictors of brain-to-brain synchrony. Rather, in the role reversal condition, the dyad’s sex make-up (specifically male–male pairs) and the participants’ perception of the relatability of their roles were identified as significant predictors. On the other hand, forward stepwise regression in the role-play condition yielded no results. Findings suggest that role-play alters interpersonal co-regulation in qualitative ways that differentiate it from typical interactions. When considering the cluster’s role in prospective memory and particularly the right lateralization of this cluster in maintaining internally generated intentions [68], it can be theorized that inhabiting another persona is related to the maintenance of intention of a second character, while at the same time the inhibition of intention of the individual themselves [32]. These internal processes are unique to role-playing. Therefore, even though behavioural outputs are expected to be qualitatively similar across conditions, behavioural indicators of synchrony are no longer able to predict brain-to-brain synchrony during role-playing conditions. Further study will be needed to confirm this conjecture.

It is also important to note that another way in which role reversal and role-play conditions differ is by the physical presence of their target character (i.e. during the role reversal condition, characters that participants are role-playing are present in the room with them, whereas they are absent during the role-play condition). This may increase the saliency of peripheral role-playing variables such as role relatability and the dyad’s sex make-up during role reversal as compared with role-play. For example, greater relatability to the character may imply a lower need for co-regulation as the participants have a stronger understanding of how to portray each other.

As for participants’ sex, it was previously found that male dyads tended to demonstrate lower behavioural synchrony than female dyads in unstructured conversations [77] and naturalistic tasks [78]. Similarly, when considering brain-to-brain synchrony, it was found that female dyads tended to show greater synchrony [79,80]. However, the present study suggests a moderating effect of role-playing conditions on dyadic sex and synchrony, where male dyads showed greater brain-to-brain synchrony in the frontal right cluster than female or mixed dyads during naturalistic conversation but lower synchrony during role reversal. While the literature generally found female dyads as having greater synchrony than female–male or male dyads, it is crucial to understand that behavioural synchrony may not translate to brain-to-brain synchrony (as is demonstrated in the present study), and synchrony in one area of the brain may not reflect synchronous activity in another. For example, Chen *et al*. [81] found that male and female dyads displayed synchrony in different areas of the brain during the same task, although the behavioural output did not differ across dyad sex. Taking into consideration the functions associated with the frontal right cluster in prospective memory, the present findings may indicate that brain-to-brain synchrony in males is more likely to be influenced by task and context demands, with complex tasks such as role reversal having a greater impact on their working memory.

To address the second research question, it appears promising that the greatest predictors of a decrease in dyad anxiety stem from role-play and role reversal experimental conditions. Specifically, during the role-play condition, a higher perceived role accuracy and a greater proportion of time spent in joint gaze were related to larger decreases in dyad anxiety. The greater perceived role accuracy may contribute to decreases in the dyad’s anxiety owing to greater confidence in their performances during the role-play condition. Additionally, findings concur with past studies that found social anxiety to be associated with gaze avoidance [82–85], possibly because eye contact activates neural circuitry related to self-conscious emotions among anxious individuals [84,85]. It, therefore, follows that the decrease in anxiety experienced within dyads is related to increased joint gaze. In fact, therapeutic interventions for anxiety disorders also use eye contact measures to evaluate the success of the treatment [86]. On the other hand, during the role reversal condition, the opposite relationship was observed for joint gaze. It is perhaps prudent then to consider the differences between role-play and role reversal conditions. During role reversal, participants witness their partner role-playing as themselves, which is a greatly vulnerable and emotionally arousing experience as compared to role-play, with practitioners claiming that it is the most potent role-play technique [31,87]. The intensity of role reversal may evoke an opposite effect among participants untrained in the method, where joint gaze negatively affected anxiety changes in the dyad. At the same time, it may also be noted that the efficacy of role reversal is dependent on the topic, as well as the similarities between the members of the dyad in question [88,89], which are factors out of the scope of this study. Finally, greater brain-to-brain synchrony in the frontal right cluster during role reversal was predictive of greater decreases in dyad anxiety. Given the cluster’s function in intention attribution and the nature of the condition, the findings suggest that dyads with high brain-to-brain synchrony are able to better discern their partner’s behaviour and intentions during the role reversal condition and relate them to the self, and as such experience lowered anxiety surrounding the interaction. This interpretation is corroborated by studies demonstrating that anxiety is related to negative self-evaluations and social situations, as well as misattributions of intent [90–92].

Results related to dyad empathy changes reveal that dyad sex is an important predictor of overall as well as empathic concern increases. As expected, female–female dyads are more likely to show increases in empathy as compared to male–male and female–male dyads. This is consistent with a body of literature suggesting that females experience greater state empathy than males, particularly in the affective domain [93–95], extending also to larger empathy changes after relevant interventions (e.g. [96]). Other significant factors that commonly appear across empathy measures include the dyad’s self-rated immersion during the role reversal. It is no surprise that greater immersion in the character is related to greater empathy for the participants’ role-playing partner, particularly as the character they are playing is their partner themselves. Although tangential, a number of studies incorporating digital tools to enhance the immersiveness of role-playing activities have shown positive results in inducing greater empathy as compared with less immersive modalities [97–99]. Additionally, a collection of behavioural synchrony measures in terms of joint vocalization and joint gaze was also identified as significant predictors of empathy change. Generally, more instances of emotional joint vocalization were predictive of greater increases in empathy, which aligns with the clinical understanding of echoing, paraphrasing or summarizing the client’s thoughts and emotions to reflect empathy [100].

### 4.1. Study limitations and future recommendations

While the study has pioneered the investigation of the relationship between neural and behavioural synchronies, there are still limitations in the study design that warrant future research and caution when interpreting the results.

First, it is often taken for granted in synchrony literature that higher synchrony necessarily translates to positive outcomes (e.g. therapeutic alliance, improved attunement and understanding). However, high synchrony as demonstrated in the present study may not translate to higher behavioural synchrony or indeed clinical outcomes. This is because the context of the social interaction needs to be taken into consideration, which was out of the scope of the present study. For example, in complex situations where complementary coordinating rather than parallel movements are needed, greater synchrony is related to poorer performance [101]. Additionally, synchrony during negatively valenced interactions such as conflict is more often than not related to poorer outcomes [102], potentially because a highly synchronous dyad will be trapped in a negative cycle [103]. In clinical contexts, it was found that clients who show non-improvement and consensual termination have the highest client–therapist synchrony [104], and vocal pitch synchrony during sessions was indicative of poorer therapeutic alliance and greater client distress [105], presumably attributable to synchrony during moments of disagreement or intense negative affect in the session. Taking this information into context, future studies may consider investigating a social interaction in smaller event windows, taking into account changes in emotional valence and other qualitative aspects to provide deeper context into the nature of the interaction. When considering role-playing techniques specifically, future studies may also wish to distinguish between moments when participants were performing in-character as compared with when they slip out-of-character (i.e. making metacommunicative comments [106]) or lengthen experimental sessions to allow participants deeper engagement in the role-play conditions. In fact, the within-subjects paradigm employed in the present study necessarily implied a shorter duration of experimental conditions to minimize participant fatigue and exposure to manipulations in the role-play technique. However, the short duration of these tasks may limit the extent of synchrony demonstrated between participant dyads, particularly when it was found that brain-to-brain synchrony increases over time in a collaborative task [107]. It should also be noted that typical clinical and psychodrama sessions are upwards of an hour long, during which interpersonal synchrony observed may fluctuate. Future studies may, therefore, consider longer task durations that more closely align with clinical interventions.

Second, the study is statistically underpowered for the analyses that were conducted, and particularly so as data from channels with bad signals also had to be discarded. However, for our purposes of exploratory research, the current findings imply a need for further investigation. Therefore, studies with larger sample sizes are required to validate the pattern of findings in the present study.

Third, owing to the technical limitations of the fNIRS, activity in the subcortical brain regions cannot be measured, and we did not consider cortical regions of the brain outside of the prefrontal cortex. While this limits the scope of inquiry into only the cortical surfaces of the brain, it must be acknowledged that other regions (e.g. temporoparietal junction) have also been implicated in brain-to-brain synchrony [108] that may be explored in future studies.

Finally, the study only included mutual friends and peers as the study sample. However, as other studies on interpersonal synchrony have revealed, there are differing degrees of synchrony observed between dyads who share different types of relationships (e.g. friends, strangers or romantic partners [42]). In the clinical context, this would include the clinician–client relationship. Future studies may consider the expansion of the current study to include more diverse profiles of participants that capture the range and complexities of relationships between the dyads.

## 5. Conclusion

While role-playing techniques are a cornerstone of psychotherapy, the implications of role-play on synchrony have not been investigated. Furthermore, there is also a dearth of studies examining the relationship between different facets of synchrony. The present study investigates the relationship between behavioural and brain-to-brain synchrony across different role-playing techniques and links them to the dyad’s reported changes in anxiety and empathy before and after the session. Results indicate that behaviour and brain-to-brain synchrony are related to each other during natural conversation but not during role-playing techniques, prompting a deeper investigation into the driving forces of interpersonal co-regulation during such unique interactions. Future studies may benefit from expanding upon the sample size, especially including a wider range of participants and relationship types. Furthermore, a more granular approach accompanied by longer condition durations may be helpful in future studies to aid the interpretation of interpersonal synchrony and enhance ecological validity with clinical and psychodramatic interventions.

## Data Availability

The datasets supporting this article have been uploaded to the Open Science Framework: [109].
